# Copolymer Engineering of Elastic–Rigid Elastomers for Wash-Durable Conductive Pastes in Wearable Textile Electronics

**DOI:** 10.3390/polym18050609

**Published:** 2026-02-28

**Authors:** Shang-Chih Chou, Yao-Yi Cheng, Jem-Kun Chen, Wilson Hou-Sheng Huang

**Affiliations:** 1 Department of Emerging Technology Research, Taiwan Textile Research Institute, New Taipei City 236003, Taiwan; 2 Institute of Organic and Polymeric Materials, National Taipei University of Technology, Taipei City 106344, Taiwan; 3 Department of Materials Science and Engineering, National Taiwan University of Science and Technology, Taipei City 106335, Taiwan; 4 Center for Continuing Education, National Tsing Hua University, Hsin Chu City 300044, Taiwan

**Keywords:** smart textiles, wearable electronics, wash durability, laundering, poly(imide-urethane) copolymer, elastomer, modulus, thermoplastic

## Abstract

Smart textiles require conductive materials that maintain electrical stability under repeated mechanical deformation and laundering while preserving textile-like flexibility. In this work, an elastic–rigid copolymer elastomer was designed as a polymer binder for washable conductive pastes used in wearable textile electronics. The copolymer was synthesized using polytetramethylene ether glycol (PTMEG), 3,3′,4,4′-benzophenonetetracarboxylic dianhydride (BTDA), and m-xylylene diisocyanate (XDI), enabling the incorporation of thermally stable imide segments and elastic polyurethane domains within a single polymer framework. By adjusting the molar ratio between rigid and soft segments, the resulting copolymer exhibited balanced tensile strength, Young’s modulus, and elastic recovery, outperforming a commercial thermoplastic polyurethane in mechanical performance. Silver-filled conductive pastes were prepared by dispersing 62 wt% micrometer-sized silver flakes into the copolymer matrix, achieving a bulk resistivity of 3.5 × 10^−5^ Ω·cm. The printed conductive films showed stable electrical resistivity under cyclic tensile deformation up to 20% strain. Washing durability was further evaluated following the AATCC 135 top-loading laundering standard. After 50 laundering cycles, the resistance increase remained within 2.8–5.5 Ω for knitted fabrics and 2.0–5.1 Ω for woven fabrics, indicating satisfactory electrical stability and adhesion to textile substrates. These results suggest that elastic–rigid copolymer binders are suitable for the development of wash-durable conductive pastes for wearable textile applications.

## 1. Introduction

In recent years, smart textiles and wearable electronics have emerged as important application fields, integrating electronic functionalities with flexible substrates to enable sensing, feedback, and communication. However, to match the actual complexities of the complex daily environment of the human body, it is necessary to develop functional clothing that can respond to a variety of human responses or feelings (e.g., strain, temperature). Constructing smart textiles with good anti-swelling performance, high sensitivity, and stable strain responses with washability applications is a great challenge. Conductive pastes used in such systems are required to simultaneously exhibit low electrical resistivity, mechanical softness, elasticity, and processing stability [1,2]. Commercial elastic conductive pastes are predominantly based on chloroprene rubber matrices filled with inorganic conductive fillers such as silver, carbon, or graphite. Some flexible and wearable electronics have accelerated research into energy storage systems that are not only lightweight and efficient but also mechanically resilient [3,4,5]. However, these materials often suffer from insufficient thermal resistance and dimensional stability during screen printing, solder-mount technology (SMT) processing, and repeated mechanical deformation.

Polyurethane (PU) and rubber are conventional flexible polymers widely used in adhesives, coatings, low-speed tires, gaskets, and cushioning materials owing to their soft molecular structures, good wear resistance, and sealing performance. However, their relatively low heat resistance and mechanical strength limit their applicability in high-temperature or mechanically demanding environments. In contrast, polyimide (PI) is a high-performance engineering polymer known for its excellent mechanical strength, chemical stability, electrical insulation, and dimensional stability over a wide temperature range. These advantages have made PI an essential material in advanced electronic and circuit board manufacturing. Nevertheless, the rigid backbone of PI significantly restricts its flexibility and elongation, thereby limiting its use in applications requiring mechanical compliance.

To integrate the complementary advantages of PU and PI, PI–PU hybrid systems have attracted increasing research interest. Kim et al. [6] reported PI–PU interpenetrating polymer networks exhibiting enhanced mechanical robustness, and PI–PU–reinforced copper foils capable of withstanding over 150 bending cycles. Al-Salah [7] further demonstrated that block-copolymerized PI–PU systems possess superior tensile performance and thermal stability compared with conventional linear PU. These studies indicate that the incorporation of rigid PI segments into flexible PU matrices offers an effective strategy to balance strength, elasticity, and thermal resistance.

Regarding synthesis strategies, Imai [8] developed a one-step polymerization route for PI via the reaction of dianhydrides and diisocyanates. Similarly, de Souza et al. [9] introduced the so-called “one-shot” or “one-pot” method for PU synthesis, in which polyols, polyethers, polyesters, and isocyanates are simultaneously introduced into a single reaction system, often with the assistance of catalysts to accelerate urethane formation. Building on these approaches, PI–PU copolymers can be synthesized through the copolymerization of dianhydrides, diol monomers, and isocyanates. In addition, 1,3,5-triazine-2,4,6-triamine (melamine) has been employed as a crosslinking agent to enhance network integrity [10], while *N*,*N*-diethylethanamine (TEA) serves as an effective catalyst to promote reactions between isocyanates and diols, thereby improving mechanical performance [9,11].

Driven by the diverse material requirements of modern industries, molecular structure design has become a key strategy for tailoring multifunctional polymer systems. Benefiting from the inherent thermal stability of PI, PI–PU films exhibit excellent temperature resistance, which effectively expands their usable temperature window [12,13,14]. Meanwhile, the flexible PU segments impart favorable ductility and elastic recovery, enabling the material to accommodate various strains and deformations. As a result, PI–PU copolymers can achieve a balanced combination of high modulus, flexibility, and thermal stability, making them promising candidates for high-performance applications.

Conventional elastomeric binders, including epoxy, waterborne cellulose, PU, and silicone, generally fail to satisfy the combined requirements of printability, thermal endurance, dielectric stability, and mechanical compliance demanded by wearable and textile-integrated electronics [15,16,17,18,19]. While PU provides excellent toughness and extensibility, it exhibits limited heat resistance and dielectric performance. Conversely, PI offers outstanding thermal stability, low dielectric loss, and dimensional integrity at elevated temperatures, albeit at the expense of flexibility and softness [20,21,22,23,24,25,26,27]. However, despite the growing interest in PI–PU copolymer systems, systematic studies that correlate copolymer composition with electromechanical stability under tensile strain and standardized laundering durability for textile-integrated conductive pastes remain limited.

**Figure 1:**
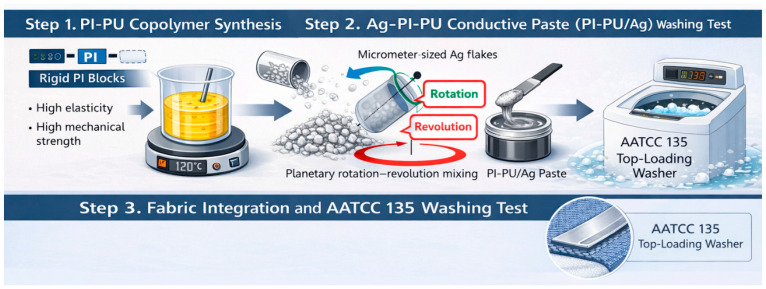
Schematic illustration of the molecular design of PI–PU copolymers, formulation of silver-filled elastic conductive pastes, and their integration into washable textile-based wearable electronic applications.

In this work, we developed a new intrinsic material of PI–PU copolymers that have emerged as promising temperature-resistant elastic binders, enabling the integration of rigid PI segments with soft PU domains to simultaneously realize thermal robustness, dielectric stability, elasticity, and controlled surface properties [24,25,26,27,28,29,30,31,32,33,34]. This elastic-rigid copolymer allows the material to undergo repeated deformations within a limited tensile range while maintaining its original electrical conductivity. Therefore, the material composition was designed to include PI as a rich phase to balance mechanical tensile strength and electrical conductivity. And the silver-filled conductive pastes based on PI–PU copolymer binders were formulated and systematically investigated [35]. Finally, the mechanical properties, electromechanical coupling behavior under strain, and laundering durability were evaluated and quantitatively benchmarked against a representative chloroprene rubber–based conductive paste. The results demonstrate the advantages of PI–PU copolymer binders for washable, mechanically reliable, and thermally stable wearable electronic applications, as schematically illustrated in Figure 1.

## 2. Experimental

### 2.1. Materials

For the synthesis of PI–PU copolymers, BTDA (Sigma-Aldrich, St. Louis, MO, USA, CAS No. 2421-28-5), XDI (Sigma-Aldrich, St. Louis, MO, USA, CAS No. 3634-83-1), and PTMEG ( Sigma-Aldrich, St. Louis, MO, USA, CAS No. 25190-06-1) were used as received unless otherwise stated. Prior to polymerization, PTMEG was dried in an oven at 80 °C for 24 h to remove residual moisture and ensure complete melting.

1-Methyl-2-pyrrolidone (NMP; Sigma-Aldrich, St. Louis, MO, USA, CAS No. 872-50-4) was used as the solvent. TEA and melamine were employed as additives. A commercial thermoplastic polyurethane (TPU; San Fan Co., Kaohsiung City, Taiwan, 70710MS) was used as a reference material. Silver flakes (Ag PRO Tech., Tainan City, Taiwan, SYP-PF 130) were used as conductive fillers for conductive paste preparation.

### 2.2. Preparation of PI–PU Polymer

PTMEG and BTDA were first introduced into a polymerization reactor and dissolved in NMP at predetermined molar ratios and solid contents. In this study, the molar ratio of PU was fixed at 1, while the molar ratio of PI was varied at 2.0, 3.25, 3.5, and 3.75, as summarized in Table 1.

The reaction mixture was stirred at 250 rpm under a nitrogen atmosphere at 75 °C until PTMEG and BTDA were completely dissolved. XDI was then gradually added to maintain the targeted stoichiometric ratio, and the mixture was allowed to react at 75 °C for 5 h. Subsequently, TEA or melamine was introduced as an additive, and the reaction temperature was increased to 95 °C.

**Figure 2:**
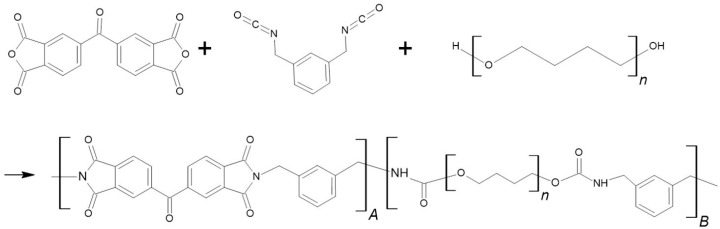
Schematic illustration of the PI–PU copolymer synthesis process.

After 30 min, the polymerization reactor was immersed in an ice-water bath (1 °C), and the stirring speed was reduced to suppress bubble nucleation. The system was then subjected to vacuum degassing for 15–60 min until no visible bubbles remained in the solution. A homogeneous PI–PU copolymer solution was thus obtained (Figure 2). The detailed compositions of the PI–PU copolymers are listed in Table 1. For example, sample N2 corresponds to a PI/PU molar ratio of 2.0.

### 2.3. Conductive Paste Preparation

Conductive pastes were prepared by dispersing 62 wt% silver flakes into the PI–PU copolymer matrix using a high-speed vacuum defoaming mixer. Mixing and degassing were completed within 6 min, 2000 rpm and −98 kPa, enabling simultaneous homogenization and removal of entrapped microbubbles between silver flakes. Compared with conventional blade mixing or three-roll milling processes, which typically require more than 3 h, this method provided a significantly faster and more efficient fabrication route.

### 2.4. Mechanical Properties

PI–PU copolymer solutions were uniformly coated onto glass substrates and dried at 80 °C for 90 min to obtain free-standing PI–PU films for mechanical testing. After cooling to room temperature, the films were carefully peeled from the substrates and cut into rectangular specimens (10 mm × 100 mm) using a laser cutter.

Tensile tests were conducted using a universal testing machine (Gotech Testing Machines Inc. Taichung City, Taiwan, TCS-2000). The specimens were tested with a gauge length of 50 mm at a strain rate of 400 mm min^−1^ until rupture or a maximum elongation of 1000%. Film thicknesses were measured individually prior to testing. For each composition, five specimens were tested, and the reported elongation values were averaged from the three most consistent results.

Elongation recovery tests were performed on specimens of identical dimensions (10 mm × 100 mm). Each specimen was mounted on a custom-built elongation recovery apparatus with an initial clamping distance of 65 mm and subjected to cyclic stretching and recovery at a rate of 65 mm s^−1^ for 100 cycles. Elongation ratios of 9%, 20%, and 80% were evaluated [36,37]. Length measurements were recorded 30 s and 1 h after unloading. Elongation recovery was calculated using Equation (1):

Elongation recovery (%) = (*L*1 − *L*1′)/(*L*1 − *L*0) × 100% (1)

where *L*0 is the original specimen length, *L*1 is the maximum length under applied strain, and *L*1′ is the length after stress release.

### 2.5. Material Structure Analysis

Fourier transform infrared (FTIR) spectra of the PI–PU films were collected using an IS50 FTIR spectrometer (Thermo Fisher Scientific, Waltham, MA, USA) in attenuated total reflection (ATR) mode. Each spectrum was obtained by averaging 32 scans over a wavenumber range of 600–4000 cm^−1^ with a resolution of 4 cm^−1^.

### 2.6. Electrical–Mechanical Stability and Resistivity Measurement

The electrical–mechanical stability of the PI–PU/Ag conductive films was evaluated under uniaxial tensile deformation. Conductive films were prepared by uniformly coating the conductive paste onto polymer substrates and curing under identical conditions prior to testing. Electrical resistance was measured using a digital multimeter in a two-probe configuration.

For strain-dependent electrical stability measurements, each specimen was stretched to a fixed strain of 20% and held for 1 min, followed by complete strain release. Resistance values were recorded immediately after unloading and after recovery times of 1, 3, and 5 min. Resistance variation was calculated relative to the initial resistance measured before deformation. This testing protocol was selected to simulate typical strain levels encountered in wearable textile applications and to assess resistance stability during cyclic deformation and recovery.

**Figure 3:**
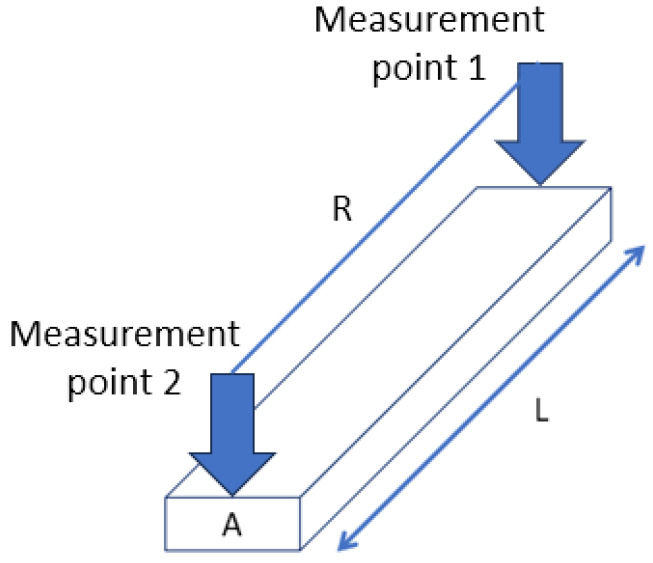
Schematic illustration of the resistivity measurement setup and sample geometry.

The volume resistivity of the printed conductive films was calculated according to Equation (2):

R = ρ (L/A)(2)

where R is the measured electrical resistance (Ω), ρ is the volume resistivity (Ω·cm), L is the conductor length (cm), and A is the cross-sectional area (cm^2^). The corresponding measurement configuration and sample geometry are schematically illustrated in Figure 3.

### 2.7. Washable/Laundry

Laundering durability is a critical requirement for smart-textile applications, as conductive materials are subjected to repeated mechanical stresses—including stretching, abrasion, friction, bending, and folding—as well as chemical exposure from detergents during washing. These combined factors are known to significantly degrade the electrical integrity of conductive pastes applied to textile substrates [38,39,40]. In this study, the laundering durability of the conductive films was evaluated in accordance with the AATCC 135 washability standard [41,42] using a top-loading washing machine, which is widely adopted to assess fabric dimensional stability and electrical reliability under repeated laundering conditions [43,44,45]. Conductive paste films were laminated onto knitted and woven textile substrates to simulate practical smart-textile configurations. Washing tests were conducted for up to 50 laundering cycles with a total load of 1.8 kg of standardized ballast fabric under controlled washing conditions. From the literature, these items can simulate the effects of other clothes inside the garment when it is being washed in a washing machine, as well as the number of times the garment is washed [46,47]. Electrical resistance was measured prior to washing and after 10, 20, 30, and 50 laundering cycles to evaluate conductivity retention. For comparison, a commercially available elastic conductive silver paste (Nagase America LLC., Itasca, IL, USA, EMS, CI-1036) was subjected to identical washing conditions. A specimen was considered to have failed when electrical continuity was lost or when the electrical resistance increased beyond the measurable range.

### 2.8. Morphological Analysis

A field emission scanning electron microscope (FE-SEM, JEOL, Peabody, MA, USA, JSM-7600F) was used to establish and analyze the correlation between the morphological observations and the electrical properties of specimens.

### 2.9. GenAI

ChatGPT (version 5.0) was used to generate the graphical abstract and schematic illustrations to enhance clarity and improve reader understanding of the manuscript, as show as Figure 1. And the computer code used is not available.

## 3. Results and Discussion

The mechanical and electrical reliability of conductive pastes for wearable textiles is strongly governed by the polymer binder, which must simultaneously provide elasticity, dimensional stability, and interfacial adhesion to textile substrates. In this study, an elastic–rigid copolymer elastomer was employed as the binder matrix to balance these competing requirements through molecular architecture design. By incorporating thermally stable imide segments as rigid domains and flexible polyurethane segments as soft domains within a single copolymer framework, the binder was expected to exhibit improved mechanical robustness while retaining sufficient elasticity for deformation under tensile strain. This copolymer design strategy was therefore systematically evaluated in terms of its mechanical properties, electromechanical stability, and washing durability when formulated as a silver-filled conductive paste for wearable textile applications.

### 3.1. FTIR Spectroscopy

**Figure 4:**
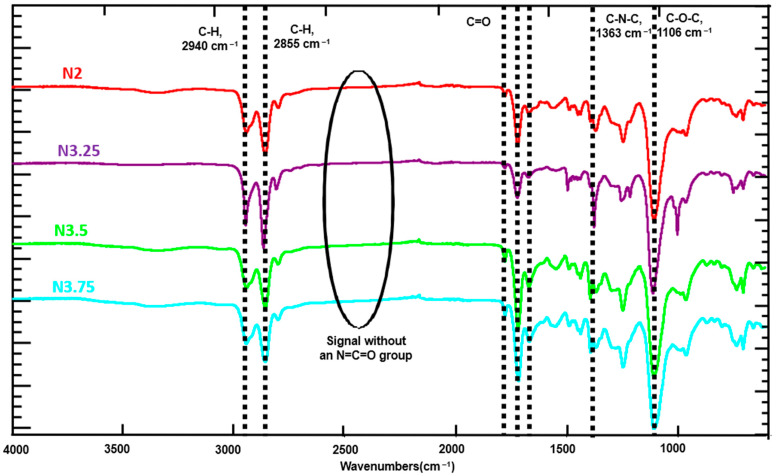
The FTIR spectra of PI–PU.

Figure 4 presents the FTIR spectra of PI–PU films with varying PI and PU contents. The complete disappearance of the isocyanate (–NCO) absorption band confirms that the polymerization reaction proceeded to completion, resulting in the successful formation of the PI–PU copolymer. For the polyimide (PI) segments, characteristic C–N–C absorption peaks were observed at 1363 cm^−1^. In addition, the typical imide carbonyl stretching vibrations appeared at 1710 and 1780 cm^−1^, corresponding to the symmetric and asymmetric C=O stretching modes of the imide groups, respectively. For the polyurethane (PU) segments, the symmetric and asymmetric stretching vibrations of the CH_2_ groups associated with ester linkages were observed at 2855 and 2940 cm^−1^, respectively [48,49,50]. The C–O–C stretching vibration was identified at 1106 cm^−1^. Moreover, the characteristic carbonyl (C=O) absorption of the urethane linkage was observed in the range of 1650–1850 cm^−1^, with a distinct band at approximately 1675 cm^−1^ attributed to hydrogen-bonded carbonyl groups. These characteristic absorption peaks collectively confirm the successful incorporation of both PI and PU segments within the PI–PU copolymer films.

### 3.2. Tensile Strength

**Figure 5:**
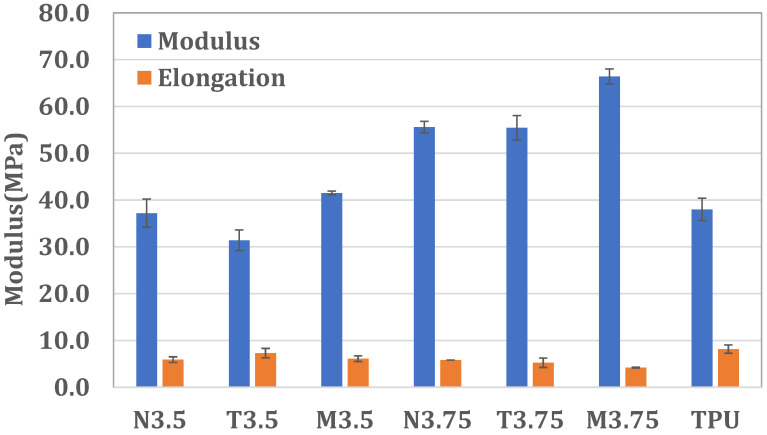
Modulus and elongation of commercial TPU and PI–PU.

When the applied strain exceeds the elastic limit, plastic deformation occurs and becomes irreversible. Accordingly, the mechanical properties of the materials were evaluated within the recoverable elongation range. To define the Hookean region for each specimen, stress–strain curves were obtained in accordance with ASTM D638, and the elastic range was identified based on the linearity of the tensile modulus. A constant slope within this region was confirmed by linear regression analysis, with coefficients of determination (R^2^) exceeding 0.99. Within the defined Hookean region, the tensile strength, tensile modulus, and elongation of PI–PU copolymers with different molar ratios were systematically compared with those of a commercial thermoplastic polyurethane (TPU) to elucidate the influence of copolymer composition on mechanical performance. The Hookean region indicates the range of tensile strength within which a material can fully recover. The results indicate that increasing the PI content leads to enhanced tensile strength and tensile modulus, reflecting increased material rigidity, while simultaneously reducing elongation. This trend highlights the reinforcing effect of rigid imide segments on the mechanical response of the copolymer. The incorporation of melamine (denoted as M) further enhances tensile strength and tensile modulus, accompanied by a reduction in elongation, indicating that melamine acts as an effective reinforcing component in the PI–PU copolymer system. In contrast, the addition of triethylamine (denoted as T) results in decreased tensile strength and modulus, while increasing elongation, suggesting improved elastic resilience due to enhanced chain mobility. As summarized in Table 2, the PI–PU film with a PI/PU molar ratio of 3.5 exhibits mechanical properties comparable to those of commercial TPU within the elastic deformation regime. As shown in Figure 5, further increasing the PI/PU molar ratio to 3.75 leads to a tensile modulus exceeding that of commercial TPU, indicating a transition toward a more rigid mechanical response. In this study, a higher proportion of PI resulted in a lower recoverable elongation but a relatively higher tensile strength, providing better electrical conductivity after stretching.

### 3.3. Elongation Recovery

**Figure 6:**
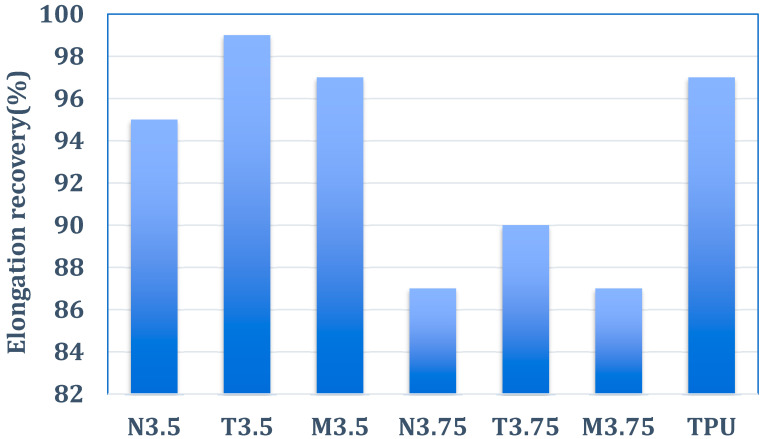
Elongation recovery after 30 s in commercial TPU and PI–PU with an elongation ratio of 9%.

As summarized in Table 3, the elongation recovery of the materials progressively decreased as the applied elongation increased from 9% to 80%, regardless of the tensile holding time (30 s or 1 h). This behavior indicates that higher deformation levels induce increased irreversible chain rearrangements, thereby reducing elastic recovery. This trend is consistent with the mechanical behavior discussed in Section 3.2, where an increase in rigid segment content led to enhanced stiffness at the expense of deformability. Moreover, increasing the BTDA molar ratio, corresponding to a higher fraction of rigid polyimide (PI) segments, resulted in a systematic reduction in elongation recovery for all PI–PU films. This observation can be attributed to the restricted segmental mobility imposed by rigid PI domains, which limits the ability of polymer chains to return to their original configurations after deformation. Such a trade-off between rigidity and elastic recovery is commonly observed in segmented copolymer systems and further supports the mechanical trends identified in the tensile analysis (Section 3.2). Notably, PI–PU membranes incorporating TEA exhibited consistently improved elongation recovery across all molar ratios. This enhancement highlights the catalytic role of TEA during polyurethane synthesis, which promotes more effective chain extension and network homogeneity within the PU phase, thereby facilitating elastic recovery under cyclic deformation. The improved resilience induced by TEA is in good agreement with the increased elongation observed in the tensile results discussed in Section 3.2. When the BTDA molar ratio was 3.5 (corresponding to a PI/PU molar ratio of 3.5), the elongation recovery behavior of the PI–PU film closely resembled that of commercial TPU, as illustrated in Figure 6. This result suggests that an optimized balance between rigid PI segments and flexible PU domains enables elastomeric performance comparable to commercial TPU while retaining the structural stability provided by the PI–PU copolymer architecture.

### 3.4. Electrical–Mechanical Stability

The M3.5 formulation exhibited superior mechanical stability among the investigated copolymer compositions and was therefore selected as the primary polymer matrix for conductive paste fabrication. The corresponding N3.5 formulation was employed as a baseline control to evaluate the influence of copolymer composition on electrical performance. In this study, the PI–PU copolymer was used as the base slurry, and silver micro-flakes were incorporated as the conductive additive to prepare PI–PU–based conductive slurries [51,52,53]. Both PI–PU/Ag conductive systems demonstrated low electrical resistivity and stable resistance under tensile deformation. As summarized in Table 4, the optimized M3.5/Ag composite achieved a low resistivity of 3.5 × 10^−5^ Ω·cm, whereas the N3.5/Ag formulation exhibited a slightly higher resistivity of 6.7 × 10^−5^ Ω·cm. Under 20% tensile strain followed by recovery, the M3.5/Ag conductor showed negligible resistance drift (0%) after recovery times of 1, 3, and 5 min. In contrast, the N3.5/Ag sample exhibited a resistance variation of up to 8.3% during the initial recovery stage, indicating less effective structural recovery of the conductive network. The significantly improved electromechanical stability of M3.5/Ag can be attributed to the balanced elastic–rigid segment architecture of the copolymer binder, which enables efficient stress redistribution within the polymer matrix while maintaining continuous conductive pathways between silver fillers. Compared with the baseline formulation, the M3.5/Ag system exhibits more stable resistance fluctuations under identical testing conditions, confirming that optimized copolymer composition plays a critical role in preserving electrical integrity under mechanical deformation. Overall, both PI–PU elastic conductive slurries (N3.5/Ag and M3.5/Ag) exhibit favorable resistance stability under tensile strain, with the M3.5/Ag formulation demonstrating superior performance. The key electrical properties of the conductive pastes are summarized in Table 4.

### 3.5. Laundering Durability

**Figure 7:**
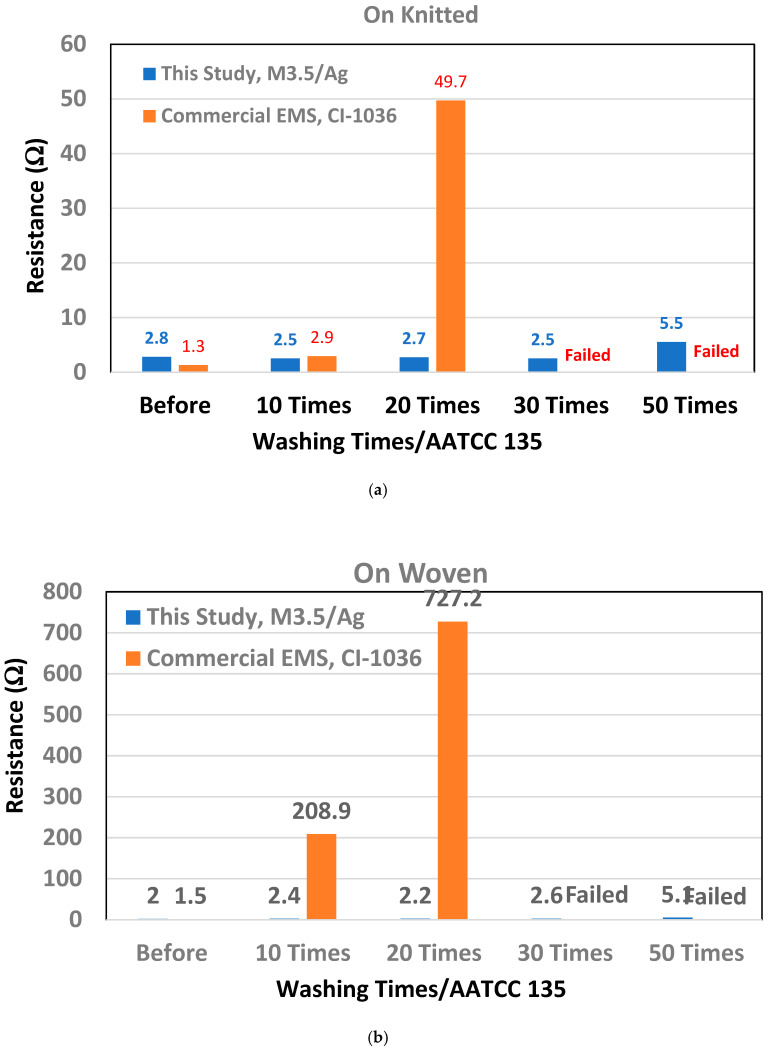
(**a**) The resistance of M3.5/Ag and CI-1036 with various washing times on the knitting. (**b**) The resistance of M3.5/Ag and CI-1036 with various washing times on the woven fabric.

To evaluate washing durability under realistic textile conditions, the prepared PI–PU/Ag conductive paste films were laminated onto knitted and woven fabric substrates. A commercially available elastic conductive silver paste/CI-1036 was included as a reference material for comparison. Laundering tests were conducted following repeated washing cycles to simulate practical smart textile applications, and the electrical resistance was monitored as a function of laundering times. The washed specimens were analyzed using resistance values. After washing, the surface of specimens may have bends and shape defects, making it impossible to accurately calculate the cross-sectional area. Therefore, resistance values are measured to analyze the electrical properties of the printed coating after washing. As summarized in Figure 7a,b, and Table 5, the M3.5/Ag conductive patterns maintained low electrical resistance throughout the washing process on both textile types. On knitted fabrics, the resistance remained below 3 Ω after 30 laundering cycles and increased moderately to 5.5 Ω after 50 cycles. Similarly, on woven fabrics, the resistance stayed below 3 Ω up to 30 cycles and reached 5.1 Ω after 50 cycles. These results indicate that the conductive networks formed by the M3.5/Ag paste remain largely intact despite repeated mechanical agitation and water exposure. In contrast, the commercial CI-1036 conductive paste exhibited rapid resistance degradation under identical laundering conditions. On knitted fabrics, the resistance increased sharply after 20 cycles and failed completely after 30 cycles. A similar failure behavior was observed on woven substrates, where resistance values exceeded 200 Ω after only 10 cycles and continued to deteriorate rapidly. This comparison highlights the superior washing durability of the PI–PU/Ag conductive system developed in this study. The enhanced laundering durability of M3.5/Ag is attributed to the intrinsic elastic recovery and mechanical robustness of the copolymer binder, which effectively accommodates washing-induced deformation while maintaining strong filler–matrix adhesion. These properties suppress crack formation and conductive pathway disruption, thereby preserving electrical functionality over extended laundering cycles. Consequently, the PI–PU/Ag conductive paste demonstrates strong potential for use in washable smart textile and wearable electronic applications.

**Figure 8:**
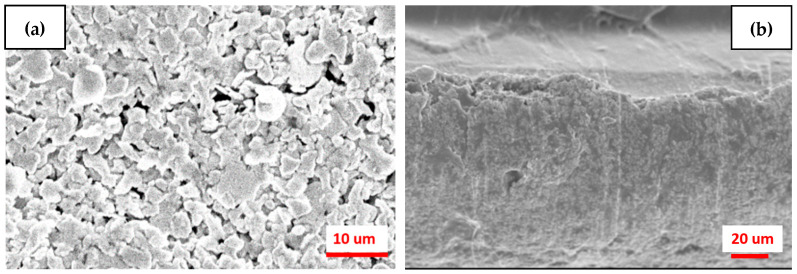
(**a**) The surface structure of this study (M3.5/Ag), (**b**) with some tiny voids between silver flakes. Figure 8b shows the cross-sectional morphology of the coating layer.

The morphological analysis of the specimen of this study (M3.5/Ag) with 50 times washing, as shown in Figure 8a,b. Figure 8a shows the surface structure of the coating layer with some tiny voids between silver flakes. Figure 8b shows the cross-sectional morphology of the coating layer. As shown in Figure 8a,b, after 50 times of washing, which brought some tiny voids to the surface, the integration of the coating layer was still complete and rigorous.

## 4. Conclusions

In this study, an elastic–rigid copolymer system was successfully developed through molecular-level design, enabling precise control over the mechanical and thermomechanical properties of elastomeric films by adjusting the ratio of rigid imide segments and flexible urethane segments. The results demonstrate that increasing the imide content effectively enhances tensile strength, while higher urethane content significantly improves elongation recovery. A clear correlation is established between elongation recovery and tensile strength, providing insight into the role of segmental mobility and chain rigidity under mechanical deformation. It is worth noting that the optimized copolymer formulation exhibits mechanical performance in the Hookean region comparable to commercial TPU during continuous stretching. When employed as a polymer binder for silver-filled conductive pastes, the copolymer enables low electrical resistivity, stable resistance under 20% cyclic strain, and excellent washing durability, as follows in the AATCC 135 standard. The melamine-modified formulation further improves mechanical robustness without sacrificing elastic recovery or electrical stability. These combined properties confirm that elastic–rigid copolymer engineering is an effective strategy for designing wash-durable, mechanically reliable conductive pastes for printed circuits in wearable and smart textile electronics.

## Figures and Tables

**Table 1:** Chemical compositions of PI–PU copolymers with different PI/PU molar ratios.

Specimens			Molar Ratio		
No Additive	TEA	Melamine	PTMEG/PU	BTDA/PI	XDI
N2	T2	M2	1	2	3.5
N3.25	T3.25	M3.25	1	3.25	4.25
N3.5	T3.5	M3.5	1	3.5	4.5
N3.75	T3.75	M3.75	1	3.75	4.75

**Table 2:** Mechanical properties of PI–PU copolymers with different molar ratios and commercial TPU evaluated within the Hookean region.

Specimens	Tensile Strength (MPa)	Young’s Modulus (MPa)	Percent Elongation (%)
N2	1.2 ± 0.0	5.8 ± 0.2	19.5 ± 0.1
T2	1.3 ± 0.1	5.7 ± 0.2	23.0 ± 0.1
M2	1.3 ± 0.0	7.2 ± 0.2	18.5 ± 0.8
N3.25	2.0 ± 0.1	28.9 ± 2.2	7.1 ± 0.5
T3.25	2.0 ± 0.2	24.8 ± 2.3	8.1 ± 0.4
M3.25	2.2 ± 0.3	30.4 ± 1.0	7.3 ± 0.7
N3.5	2.2 ± 0.3	37.2 ± 3.0	5.9 ± 0.6
T3.5	2.3 ± 0.3	31.4 ± 2.2	7.3 ± 1.0
M3.5	2.6 ± 0.1	41.5 ± 0.4	6.1 ± 0.6
N3.75	3.4 ± 0.3	55.6 ± 1.2	5.8 ± 0.0
T3.75	2.8 ± 0.5	48.9 ± 2.6	5.8 ± 1.0
M3.75	2.7 ± 0.0	66.4 ± 1.6	4.2 ± 0.1
TPU/Commercial	3.0 ± 0.0	38.0 ± 2.4	8.2 ± 0.9

**Table 3:** Elongation recovery of commercial TPU and PI–PU.

	30 s			1 h		
Specimens	9%	20%	80%	9%	20%	80%
N2	100	96	96	100	99	99
T2	100	100	97	100	99	99
M2	100	98	96	100	100	99
N3.25	98	92	89	99	95	94
T3.25	97	94	91	100	98	96
M3.25	97	92	89	98	98	94
N3.5	95	94	88	98	97	94
T3.5	99	94	88	100	97	95
M3.5	97	90	87	100	96	93
N3.75	87	85	82	94	93	90
T3.75	90	94	86	98	96	92
M3.75	87	86	81	96	94	90
TPU/Commercial	97	98	95	100	100	97

**Table 4:** Electrical resistivity and resistance variation in PI–PU/Ag conductive slurries after 20% tensile strain.

	Resistivity (Ω·cm)	Resistance Variation Rate (%)		
		Recovery Times (min)		
		1	3	5
N3.5/Ag	6.7 × 10^−5^	8.3	8.3	0
M3.5/Ag	3.5 × 10^−5^	0	0	0

**Table 5:** Electrical resistance of conductive patterns on knitted and woven fabrics after repeated laundering (AATCC 135).

Conductive Paste	Textiles	Before	10 Times	20 Times	30 Times	50 Times
This Study, M3.5/Ag	Knitting	2.8	2.5	2.7	2.5	5.5
This Study, M3.5/Ag	Woven	2.0	2.4	2.2	2.6	5.1
Commercial EMS, CI-1036	Knitting	1.3	2.9	49.7	Failed	Failed
Commercial EMS, CI-1036	Woven	1.5	208.9	727.2	Failed	Failed

## Data Availability

The original contributions presented in this study are included in the article. Further inquiries can be directed to the corresponding authors.
